# Microdosimetric analysis of ^211^At in thyroid models for man, rat and mouse

**DOI:** 10.1186/2191-219X-2-29

**Published:** 2012-06-09

**Authors:** Anders Josefsson, Eva Forssell-Aronsson

**Affiliations:** 1 Department of Radiation Physics, Institute of Clinical Sciences, Sahlgrenska Cancer Center, Sahlgrenska Academy at the University of Gothenburg, Sahlgrenska University Hospital, Gothenburg, 413 45, Sweden

**Keywords:** microdosimetry, ^211^At, thyroid follicle, specific energy, Monte Carlo

## Abstract

**Background:**

The alpha particle emitter ^211^At is proposed for therapy of metastatic tumour disease. ^211^At is accumulated in the thyroid gland in a similar way as iodine. Dosimetric models of ^211^At in the thyroid are needed for radiation protection assessments for 1) patients receiving ^211^At-labelled pharmaceuticals where ^211^At may be released *in vivo* and 2) personnel working with ^211^At. Before clinical trials, preclinical studies are usually made in mice and rats. The aims of this study were to develop thyroid models for mouse, rat and man, and to compare microdosimetric properties between the models.

**Methods:**

A thyroid follicle model was constructed: a single layer of 6 to 10-μm thick follicle cells with centrally positioned 4 to 8 μm (diameter) spherical nuclei surrounded a 10 to 500 μm (diameter) spherical follicle lumen. Species-specific models were defined for mouse, rat and man. The source compartments for ^211^At were the follicle lumen, follicle cells and follicle cell nuclei. The target was the follicle cell nucleus. Simplified species-specific thyroid models were used to investigate the contribution from surrounding follicles. Monte Carlo simulations were performed using the general purpose radiation transport code MCNPX 2.6.0.

**Results:**

When ^211^At was homogeneously distributed within the follicle lumen, the mean specific energies per decay, 〈z〉, to the follicle cell nucleus were 2.0, 1.1 and 0.17 mGy for mouse, rat and man, respectively. Corresponding values for the single-hit mean specific energy per decay, 〈z_1_〉, were 1.3, 0.61 and 0.37 Gy. Assuming a homogeneous ^211^At concentration in the follicle lumen, <0.5%, 7%, and 45% of the emitted alpha particles were fully stopped within the follicle lumen for the respective models.

**Conclusions:**

The results clearly show the influence of the follicle size, alpha particle range and ^211^At location within the thyroid follicle on the dosimetric parameters. Appropriate thyroid models are required for translation of dosimetric parameters between species.

## Background

The alpha emitter ^211^At is proposed in radionuclide therapy because of the optimal linear energy transfer (LET), high RBE value and independence of dose rate and oxygen level [1,2]. ^211^At is well suited for therapy of microscopic tumours due to the short range of the alpha particles, 48 to 70 μm in liquid water [3]. ^211^At decays through two branches both with the emission of an alpha particle to stable ^207^Pb, and in the decay process, 77 to 92 keV K X-rays are emitted, permitting external detection and imaging [4,5]. Astatine is like iodine a halogen, and free ^211^At is taken up by the thyroid gland partly in a similar way as iodide. *In vitro* studies have shown that the transport of ^211^At in the basal-to-apical direction was partly similar to that of ^125^I, although differences occur [6].

Relatively, few studies have been performed of the pharmacokinetics of free ^211^At in animals and man, most of them 60 to 70 years ago [7-15]. The data show a high uptake in normal thyroid gland of ^211^At in monkeys, guinea pigs, rats and mice. The maximum uptake has been reported to occur at approximately 4 h after injection for mice [12,13].

For radiation protection purposes it is, thus, important to have appropriate dosimetric models of ^211^At in the thyroid. Such models are valuable both for patients receiving ^211^At-labelled pharmaceuticals, where some ^211^At might be released *in vivo,* and also for personnel handling ^211^At with a potential risk of internal contamination. Furthermore, preclinical experimental therapeutic studies of ^211^At-labelled substances are usually performed on mice and rats before possible introduction to the clinic. Then, methods to translate data between animals and man are of great value to estimate absorbed dose to critical organs and predict side effects.

The aims of this study were to develop thyroid models for mouse, rat and man, and to perform Monte Carlo simulations for ^211^At to study the effect of geometry and biodistribution on microdosimetric parameters for the three species.

## Methods

Two different models were developed in this work: 1) a single follicle model and 2) a multiple thyroid follicle model.

### Single thyroid follicle model

**Figure 1:**
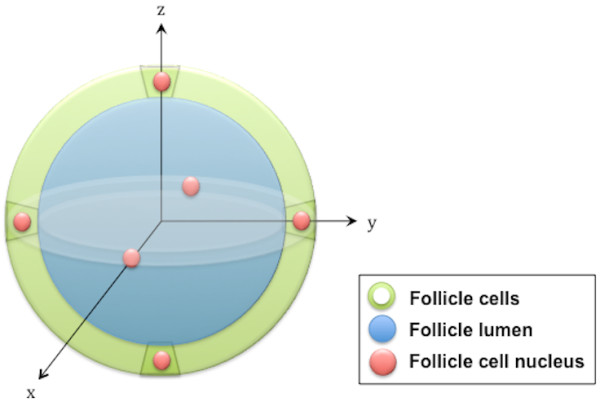
**Schematic illustration of the single thyroid follicle model.** Schematic illustration of the spherical thyroid follicle geometry; a follicle lumen (blue) is surrounded by a layer of follicle cells (green) with six symmetrically positioned follicle cell nuclei (red) from which an average value for a follicle cell nucleus is calculated.

The single thyroid follicle model used in this work consists of a single layer of thyroid follicular cells surrounding a spherical follicle lumen (Figure 1). The follicle lumen diameter was 10 to 500 μm. The surrounding follicle cells had a thickness of 6, 8 or 10 μm with a centrally located spherical nucleus with diameters of 4, 6 and 8 μm, respectively. For the species-specific models, the follicle lumen diameter, follicle cell thickness and nucleus diameter were: 1) 50, 6 and 4 μm for mouse, 2) 70, 8 and 6 μm for rat and 3) 150, 10 and 8 μm for man, respectively. The densities of the follicle lumen and the follicular cells were assumed to be that of liquid water (unit density). The selected dimensions were chosen according to the literature and further presented in the ‘Discussion’ section [16-23].

Four different source compartments and ^211^At distributions were used: A) ^211^At homogeneously distributed within the follicle lumen, B) ^211^At distributed on concentric spherical surfaces within the follicle lumen (to investigate heterogeneous distributions within the follicle lumen), C) ^211^At homogeneously distributed within the follicle cells and D) ^211^At homogeneously distributed within the follicle cell nuclei. The follicle cell nuclei were the target in most simulations. The six follicular cell nuclei symmetrically positioned on the perpendicular *x*-, *y*- and *z*-axes around the follicle lumen were used to calculate an average value for one nucleus.

### Multiple thyroid follicle model

**Figure 2:**
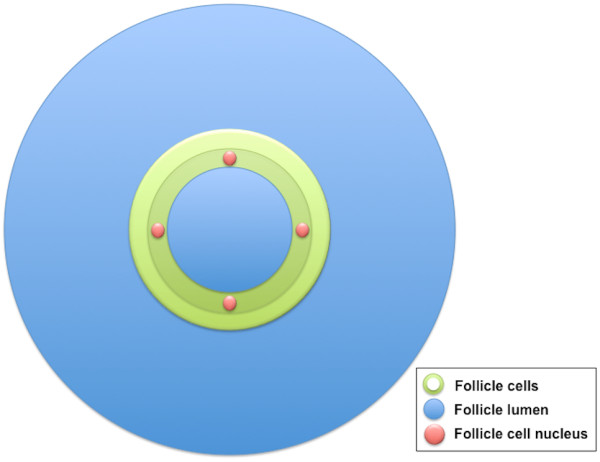
**Schematic illustration of the multiple thyroid follicle model.** Schematic illustration for one spherical thyroid follicle surrounded by neighbouring follicles. In the centre, a follicle lumen (blue) is surrounded by follicle cells (green) with centrally placed follicle cell nuclei (red). The follicle is surrounded with a single layer of neighbouring follicle cells (green) and follicle lumen (blue).

The model was used to calculate the contribution from one layer of neighbouring follicles to the follicle cell nuclei in a centrally placed follicle (Figure 2). Calculations were performed for the mouse, rat and human models. The neighbouring follicles were modelled as one surrounding layer of follicle cells and an outer region simulating the follicle lumens with the respective thicknesses: 1) 6 and 50 μm for the mouse, 2) 8 and 70 μm for the rat, and 3) 10 and 150 μm for the human model. In this model, two source compartments were used: E) ^211^At homogeneously distributed within the surrounding follicle lumens, and F) ^211^At homogeneously distributed within the surrounding follicle cells. The targets were the follicle cell nuclei in the central follicle, and the six symmetrically positioned follicular cell nuclei were used to calculate an average value for one nucleus.

### Monte Carlo simulations

The simulations were performed using the general purpose Monte Carlo radiation transport code MCNPX 2.6.0 [24] on a MacBook Pro with a 2.66 GHz Intel Core 2 Duo processor with Mac OS X version 10.6.8 (Apple Inc., Cupertino, California, United States). The tally F6, in units of MeV/g, was used in MCNPX to calculate the energy deposited in the target volume. The cut-off energy for the simulated alpha particles was 1 keV, and the sampling frequency was the default value in the MCNPX code. For each geometric setup, a number of 10^7^ particle histories were simulated, and one history represented a single decay of ^211^At. Only the main two alpha particle energies in the ^211^At decay, i.e. 5.867 MeV (42% yield) and 7.450 MeV (58% yield), were used in the simulations. The photons and electrons emitted were not considered since their energy deposits in the target is negligible compared to that of the alpha particles. The recoil energy from the daughter nuclei ^207^Bi and ^207^Pb following the alpha decay, with the kinetic energy of 114 and 144 keV, respectively [25], was also not considered since it would only contribute for ^211^At distributed within the follicle cell nucleus, maybe a less likely situation. The total decay of ^211^At to ^207^Pb was assumed to occur in the same volume element, i.e. diffusion of ^211^Po (0.52 s half-life) before alpha decay was neglected.

To investigate the effects on the microdosimetric parameters for different concentrations of stable iodine within the follicle lumen, additional calculations were performed for a 1% and 2% concentrations of stable iodine (^127^I, density 4.94 g/cm^3^) homogeneously distributed within the follicle lumen.

### Microdosimetric parameters

The following microdosimetric parameters were simulated in this work: the mean specific energy, 〈z〉, is the average energy deposited in the target per decay of ^211^At, including histories with zero specific energy

(1) $$\langle z\rangle =\frac{1}{N}\sum_{i=1}^{N}z_{i}\text{,}\left(z_{i}\geq 0\right)\text{,}\left[Gy\right]\text{,}$$

and is equivalent to the S-value in the MIRD formalism [26]. *N* is the total number of histories simulated, and *z*_*i*_, the specific energy in units of Gy for history *i*[27].

The single-hit mean specific energy per event, 〈z_1_〉, is the average energy deposited for the histories when the particle hits the target, i.e. excluding all histories with zero specific energy

(2) $$\langle z_{1}\rangle =\frac{1}{M}\sum_{i=1}^{M}z_{i}\text{,}\left(z_{i}>0\right)\text{,}\left[Gy\right]\text{,}$$

where *M* is the total number of histories simulated that hits the target [27]. The single-hit specific energy distribution for the target is denoted as *f*(*z*_1_).

The lineal energy, *y*, is defined as

(3) $$y=\frac{\varepsilon }{\overset{?¯}{l}}\text{,}\left[keV/\mu m\right]\text{,}$$

where *ε* is the energy imparted in the target volume and $\overset{?¯}{l}$ is the mean chord length for the target [27]. Based on Cauchy’s theorem, the mean chord length for a spherical volume with the diameter, *d*, is given by [28]

(4) $$\overset{?¯}{l}=\frac{2\cdot d}{3}\text{,}\left[\mu m\right]$$

The frequency-mean lineal energy, $\langle y_{F}\rangle$, is the average energy deposited for all simulated particles that hit the target

(5) $$\langle y_{F}\rangle =\frac{1}{M}\sum_{i=1}^{M}y_{i}\text{,}\left(y_{i}>0\right)\text{,}\left[keV/\mu m\right]$$

The lineal energy distribution, *f*(*y*), is defined for single energy-deposition events only and is independent of the absorbed dose or dose rate [27].

### Macrodosimetry

According to the MIRD formalism, the mean absorbed dose, $\overset{?¯}{D}$, is the average energy per unit mass deposited in a target volume

(6) $$\overset{?¯}{D}=\tilde{A}\cdot \Delta \cdot \frac{\phi }{m}\text{,}\left[Gy\right]\text{,}$$

where *Ã* is the cumulated activity in the source volume, Δ the mean energy emitted per nuclear transition, and *ϕ* is the absorbed fraction in the thyroid and *m* the mass of the thyroid [26].

The mean absorbed dose could also be expressed as

(7) $$\overset{?¯}{D}=\tilde{A}\cdot S\text{,}\left[Gy\right]\text{,}$$

where *S* is equivalent to the mean specific energy, 〈z〉.

When comparing MIRD and microdosimetric data, the ^211^At activity in the thyroid gland was assumed to be homogeneously distributed within the follicle lumens, and 70% of the thyroid gland was assumed to consist of follicles in the microdosimetric calculations [29].

The cumulated activity equivalent to a mean absorbed dose to the thyroid of 1 Gy for the different species was calculated under the following assumptions: *ϕ* = 1, Δ = 6.8 MeV and *m* is 3 mg for mouse, 30 mg for rat and 19 g for man [12,17,30,31].

### Statistical analyses

The relative error, which is the ratio between the standard deviation of the mean value (SD) and the mean value, was calculated using MCNPX [32].

## Results

### Single thyroid follicle model with ^211^At distributed in the follicle lumen

#### *Mean specific energy, 〈z〉*

**Figure 3:**
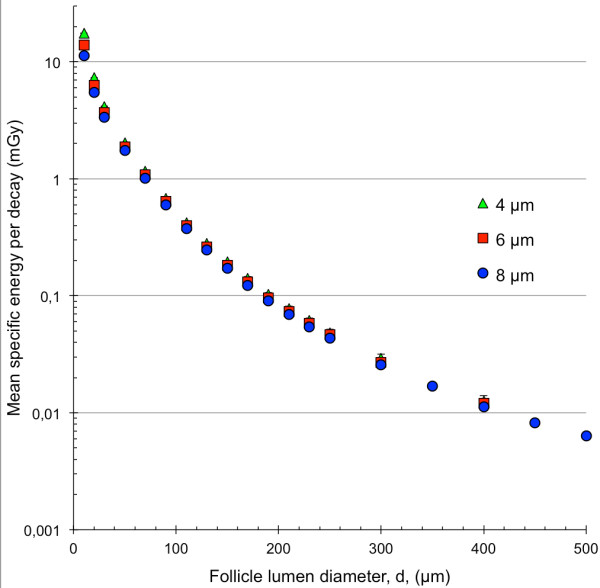
**Mean specific energy for the single thyroid follicle model.** The mean specific energy, 〈z〉, for the 8- (blue filled circle), 6- (red filled square) and 4-μm (green filled triangle) follicle cell nucleus per decay. ^211^At was homogeneously distributed within the follicle lumen. The follicle lumen diameter, *d*, varied between 10 and 500 μm. Note the logarithmic scale on the *y*-axis. Error bars indicate SD and are smaller than the data symbol when not visible.

The mean specific energy, 〈z〉, for the 4-, 6- and 8-μm diameter follicle cell nuclei with ^211^At homogeneously distributed within the follicle lumen as a function of the follicle lumen diameter is shown in Figure 3. For the 10-μm diameter follicle lumen diameter, 〈z〉 was 35% higher for the 4-μm compared with the 8-μm diameter follicle cell nuclei. This difference decreased to about 12% for the 70-μm follicle lumen diameter and remained constant at around 12% to 13% for increasing follicle lumen diameter.

**Figure 4:**
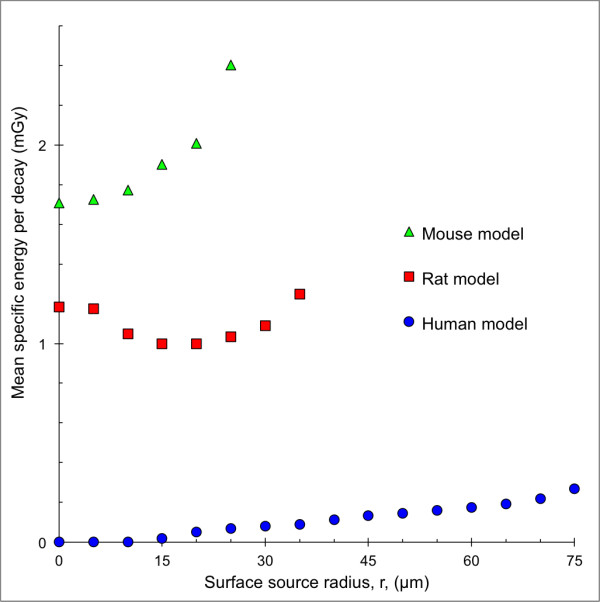
**Mean specific energy for the species-specific thyroid follicle models.** The mean specific energy, 〈z〉, for the human (blue filled circle), rat (red filled square) and mouse models (green filled triangle) per decay. ^211^At was homogeneously distributed on concentric spherical surfaces with radius, *r*, within the follicle lumen. For the 0-μm radius surface, a point source was used. Error bars indicate SD and are smaller than the data symbol when not visible.

The mean specific energy, 〈z〉, for the mouse, rat and human models with ^211^At homogeneously distributed on concentric spherical surfaces within the follicle lumen as a function of the surface source radius is shown in Figure 4. 〈z〉 was highest for the mouse model and lowest for the human model. For the human and mouse models, 〈z〉 increased with the surface source radius to a maximum peripherally in the follicle lumen. For the rat model, 〈z〉 initially decreased with increasing surface source radius, reaching a minimum for a radius of 15 μm, then increasing to a maximum peripherally in the follicle lumen.

#### *Single-hit mean specific energy,*〈z_1_〉

**Figure 5:**
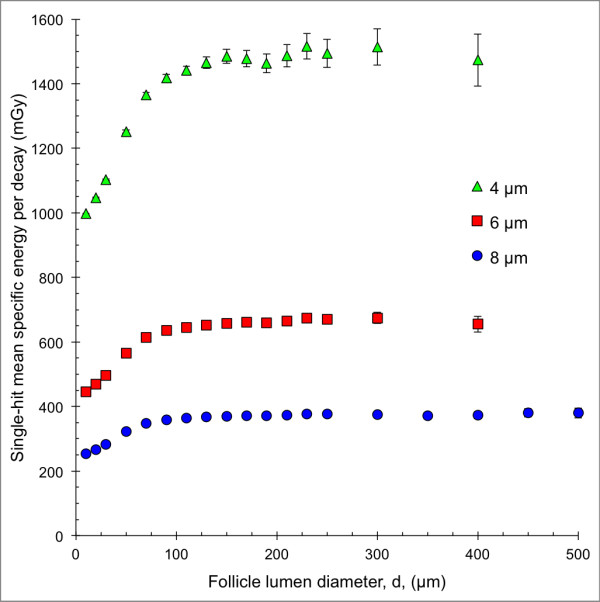
**Single-hit mean specific energy for the single thyroid follicle model.** The single-hit mean specific energy, 〈z_1_〉, for the 8- (blue filled circle), 6- (red filled square) and 4-μm (green filled triangle) follicle cell nucleus per decay. ^211^At was homogeneously distributed within the follicle lumen. The follicle lumen diameter, *d*, varied between 10 and 500 μm. Error bars indicate SD and are smaller than the data symbol when not visible.

The single-hit mean specific energy, 〈z_1_〉, for the 4-, 6- and 8-μm diameter follicle cell nuclei with ^211^At homogeneously distributed within the follicle lumen as a function of the follicle lumen diameter is shown in Figure 5. 〈z_1_〉 initially increased with the follicle lumen diameter up to a level after which it remained constant. This level was reached at the follicle lumen diameters of 150-, 110- and 90-μm for the follicle cell nuclei diameters 4, 6 and 8 μm, respectively. 〈z_1_〉 for the 4-μm nucleus diameter was about two to four times higher than for the 6-μm and 8-μm follicle cell nucleus diameters, respectively.

**Figure 6:**
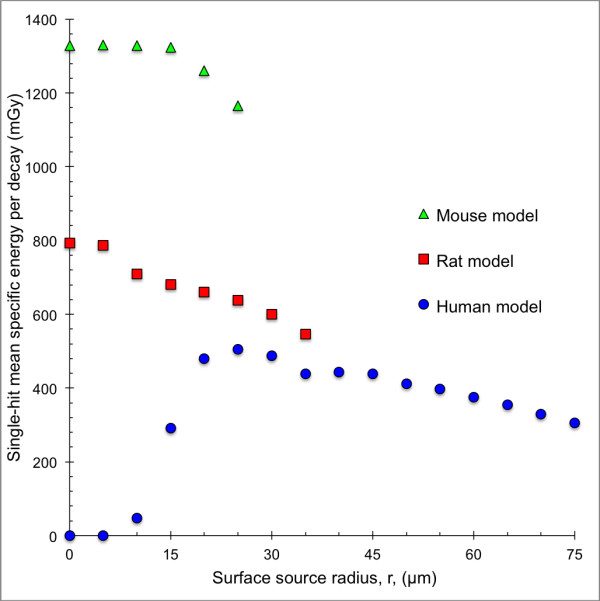
**Single-hit mean specific energy for the species-specific thyroid follicle models.** The single-hit mean specific energy, 〈z〉, for the human (blue filled circle), rat (red filled square) and mouse model (green filled triangle) per decay. ^211^At was homogeneously distributed on concentric spherical surfaces with radius, *r*, within the follicle lumen. For the 0-μm radius surface, a point source was used. Error bars indicate SD and are smaller than the data symbol when not visible.

The single-hit mean specific energy, 〈z_1_〉, for the mouse, rat and human models with ^211^At homogeneously distributed on concentric spherical surfaces within the follicle lumen as a function of the surface source radius is shown in Figure 6. 〈z_1_〉 was highest for the mouse model and lowest for the human model. For the mouse and rat models, 〈z_1_〉 decreased with increasing surface source radius from a maximum value in the centre of the follicle lumen. The minimum value was reached peripherally in the follicle lumen for the largest surface source radius. The human model had the minimum value of 〈z_1_〉 = 0 in the centre of the follicle lumen when the alpha particles did not reach the follicle cell nuclei. The maximum 〈z_1_〉 value was reached for the surface source radius 25 μm; after which; it decreased with increasing surface source radius.

#### *Single-hit specific energy distribution, f(z*_*1*_*)*

**Figure 7:**
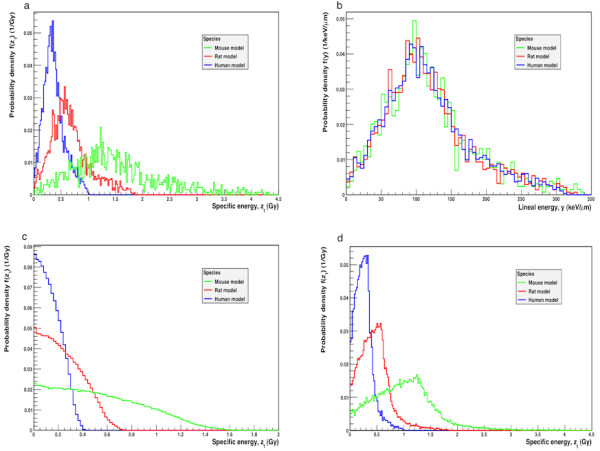
**Probability distributions for the art-specific thyroid follicle models.** (**a**) Single-hit specific energy distribution, *f*(*z*_1_), for the mouse, rat and human models per ^211^At decay that hits the target nuclei with ^211^At homogeneously distributed within the follicle lumen. (**b**) Lineal energy distribution, *f*(*y*), for the mouse, rat and human models per ^211^At decay that hits the target nuclei with ^211^At homogeneously distributed within the follicle lumen. (**c**) Single-hit specific energy distribution, *f*(*z*_1_), for the mouse, rat and human models with ^211^At homogeneously distributed within the follicle cell nucleus. This is the self-absorbed specific energy distribution for the follicle cell nucleus. For panels a, c and d, the bin size used were 20 mGy; for panel b, the bin size used was 5 keV/μm, and the area under each curve was normalised to 1. (**d**) Single-hit specific energy distribution, *f*(*z*_1_), for the mouse, rat and human models per ^211^At decay that hits the target nuclei with ^211^At homogeneously distributed within the follicle cells.

The single-hit distribution of specific energy, *f*(*z*_1_), in the follicle cell nucleus for the mouse, rat and human models with ^211^At homogeneously distributed within the follicle lumen is shown in Figure 7a. The maximum single-hit specific energies were 4.4, 1.9 and 0.99 Gy, respectively, for mouse, rat and man. The corresponding single-hit mean specific energies, 〈z_1_〉, were 1.3, 0.61 and 0.37 Gy, respectively, for mouse, rat and man (Table 1).

**Table 1:** 〈z〉 **and** 〈z_1_〉 **data for single thyroid follicle models depend on source compartment**

	**Nucleus ← Nucleus**	**Nucleus ← Follicle cells**		**Nucleus ← Lumen**	
	〈z〉 **and** 〈z_1_〉 **(Gy)**	〈z〉 **(Gy)**	〈z_1_〉 **(Gy)**	〈z〉 **(Gy)**	〈z_1_〉 **(Gy)**
Mouse	5.36E-1	2.74E-3	1.01E0	2.03E-3	1.25E0
	(1.13E-4)	(1.91E-5)	(3.48E-3)	(1.76E-5)	(4.67E-3)
Rat	2.40E-1	1.40E-3	4.60E-1	1.08E-3	6.14E-1
	(5.07E-5)	(9.38E-6)	(1.61E-3)	(9.12E-6)	(2.33E-3)
Man	1.36E-1	3.17E-4	2.57E-1	1.71E-4	3.70E-1
	(2.86E-5)	(3.36E-6)	(1.42E-3)	(2.83E-6)	(2.79E-3)

The mean specific energy, 〈z〉, and the single-hit mean specific energy, 〈z_1_〉 for the mouse, rat and human models per decay. ^211^At was homogeneously distributed within the source volume (nucleus, follicle cells or lumen), and the target was the follicle cell nucleus. Note: data are given as mean (SD).

#### *Lineal energy distribution, f(y)*

The lineal energy distribution, *f*(*y*), in the follicle cell nucleus for the mouse, rat and human models with ^211^At homogeneously distributed within the follicle lumen, is shown in Figure 7b. The maximum lineal energies were 340, 330 and 310 keV/μm, respectively, for mouse, rat and man. The frequency-mean lineal energy, $\langle y_{F}\rangle$, for mouse was 98 keV/μm, for rat 110 keV/μm, and for man 120 keV/μm.

#### *Fraction of the emitted alpha particles that deposit all energy in the follicle lumen*

**Figure 8:**
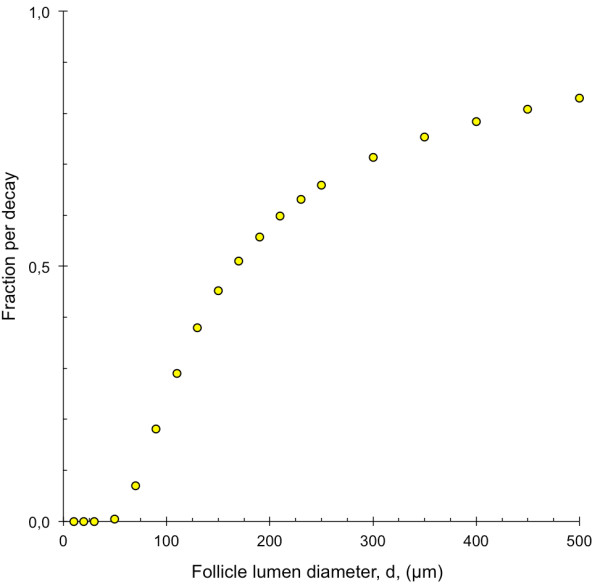
**Fraction of emitted alpha particles that deposit all kinetic energy within the follicle lumen.** The fraction of emitted alpha particles per decay that deposited all kinetic energy within the follicle lumen for follicle lumen diameter, *d*, between 10 and 500 μm. ^211^At was homogeneously distributed within the follicle lumen. Error bars indicate SD and are smaller than the data symbol when not visible.

The fraction of the total emitted alpha particles per decay that deposit all kinetic energy within the follicle lumen, with ^211^At homogeneously distributed within the follicle lumen, is shown in Figure 8. For follicle lumen diameters smaller than 50 μm, less than 0.5% deposits all kinetic energy within the lumen. For a diameter of 170 μm, the contribution from the two different alpha particles emitted per decay is equal, 25.5% each, resulting in a total of 51% that deposits all kinetic energy within the lumen. For follicle lumen diameters smaller than 170 μm, the contribution from the alpha particle with the lower kinetic energy (5.867 MeV) dominates, and for larger follicle lumen diameters, the contribution from the alpha particle with the higher kinetic energy (7.45 MeV) dominates. For the species-specific models, the amounts of alpha particles depositing all their energy in the follicle lumen were <0.5%, 7% and 45% for mouse, rat and man, respectively.

### Single thyroid follicle model with ^211^At distributed in the follicle cells

#### *Specific energy distribution, f(z), for*^*211*^*At distributed in the follicle cell nucleus*

The specific energy distribution, *f*(*z*), for ^211^At homogeneously distributed within in the different sized cell nuclei is shown in Figure 7c. For each of the models, mouse, rat and man, 〈z〉 and 〈z_1_〉 and are equal since the nuclei are both sources and targets and were 0.54, 0.24 and 0.14 Gy, respectively (Table 1).

#### *Single-hit specific energy distribution, f(z*_*1*_*), for*^*211*^*At distributed in the follicle cells*

The single-hit distribution of specific energy, *f*(*z*_1_), for the mouse, rat and human models with ^211^At homogeneously distributed within the follicle cells, including both cytoplasm and nucleus, is shown in Figure 7d. For the mouse, rat and human models, 〈z〉 were 2.7, 1.4, 0.32 mGy, and 〈z_1_〉 were 1.0, 0.46 and 0.26 Gy, respectively (Table 1).

### Influence of stable iodine in the follicle lumen for a single follicle model

Table 2 shows 〈z〉 and 〈z_1_〉 for the mouse, rat and human models with concentrations of 0%, 1% and 2% of stable iodine, ^127^I, homogeneously distributed within the follicle lumen. For the mouse model, there was a small increase in 〈z〉 with increasing iodine concentration. For the rat and human models, the opposite result was found. For the mouse and rat models, there was a small increase in 〈z_1_〉, while in the human model, 〈z_1_〉 remained fairly constant with increasing iodine concentration.

**Table 2:** **Influence of stable iodine on** 〈z〉 **and** 〈z_1_〉 **in the single thyroid follicle models**

	**0% Iodine**		**1% Iodine**		**2% Iodine**	
	**Nucleus ← Lumen**		**Nucleus ← Lumen**		**Nucleus ← Lumen**	
	〈z〉 **(Gy)**	〈z_1_〉 **(Gy)**	〈z〉 **(Gy)**	〈z_1_〉 **(Gy)**	〈z〉 **(Gy)**	〈z_1_〉 **(Gy)**
Mouse	2.03E-3	1.25E0	2.04E-3	1.26E0	2.04E-3	1.27E0
	(1.76E-5)	(4.67E-3)	(1.78E-5)	(4.73E-3)	(1.79E-5)	(4.83E-3)
Rat	1.08E-3	6.14E-1	1.08E-3	6.18E-1	1.07E-3	6.21E-1
	(9.12E-6)	(2.33E-3)	(9.13E-6)	(2.35E-3)	(9.12E-6)	(2.42E-3)
Man	1.71E-4	3.70E-1	1.66E-4	3.70E-1	1.61E-4	3.70E-1
	(2.83E-6)	(2.79E-3)	(2.79E-6)	(2.86E-3)	(2.75E-6)	(2.91E-3)

The mean specific energy, 〈z〉, and the single-hit mean specific energy, 〈z_1_〉, for the mouse, rat and human models per ^211^At decay. The follicle lumen consisted of water and homogeneously distributed stable iodine (0%, 1% or 2% ^127^I). ^211^At was homogeneously distributed within the follicle lumen, and the target was the follicle cell nucleus. Note: data are given as mean (SD).

### Multiple thyroid follicle models

The contribution from ^211^At homogeneously distributed within one surrounding layer of neighbouring follicles to a central-placed follicle for the mouse, rat and human models is presented in Table 3. The contribution to 〈z〉 from one ^211^At decay in the surrounding follicle lumen was low, only 13%, 7% and 4% for mouse, rat and man, respectively, compared to that from one ^211^At decay in the actual follicle lumen. Corresponding values for 〈z_1_〉 are 130%, 120% and 110%, demonstrating that 〈z_1_〉 from ^211^At in surrounding follicle lumen was higher than from the actual follicle. For ^211^At homogeneously distributed within the surrounding follicle cells, 〈z〉 was up to half of the values for ^211^At homogeneously distributed within the central follicle cells per decay. Corresponding values for 〈z_1_〉 were up to 130%, demonstrating higher values for ^211^At homogeneously distributed within the surrounding follicle cells per decay (Table 3).

**Table 3:** 〈z〉 **and** 〈z_1_〉 **data for the single and multi-thyroid follicle models**

	**Single follicle**				**Surrounding follicles**			
	**Nucleus ← Lumen**		**Nucleus ← Follicle cells**		**Nucleus ← Lumen**		**Nucleus ← Follicle cells**	
	〈z〉 **(Gy)**	〈z_1_〉 **(Gy)**	〈z〉 **(Gy)**	〈z_1_〉 **(Gy)**	〈z〉 **(Gy)**	〈z_1_〉 **(Gy)**	〈z〉 **(Gy)**	〈z_1_〉 **(Gy)**
Mouse	2.03E-3	1.25E0	2.74E-3	1.01E0	2.57E-4	1.67E0	1.50E-3	1.26E0
	(1.76E-5)	(4.67E-3)	(1.91E-5)	(3.48E-3)	(7.39E-6)	(2.22E-2)	(1.54E-5)	(5,77E-3)
Rat	1.08E-3	6.14E-1	1.40E-3	4.60E-1	7.60E-5	7.37E-1	7.38E-4	5.86E-1
	(9.12E-6)	(2.33E-3)	(9.38E-6)	(1.61E-3)	(2.65E-6)	(1.16E-2)	(7.38E-6)	(2.67E-3)
Man	1.71E-4	3.70E-1	3.17E-4	2.57E-1	6.24E-6	4.13E-1	1.74E-4	3.37E-1
	(2.83E-6)	(2.79E-3)	(3.36E-6)	(1.42E-3)	(5.73E-7)	(1.77E-2)	(2.71E-5)	(2.36E-3)

The mean specific energy, 〈z〉, and the single-hit mean specific energy, 〈z_1_〉, for the mouse, rat and human models per decay. ^211^At was homogeneously distributed within the follicle lumens and follicle cells, and the target was the follicle cell nucleus in the single/central follicle. Note: data are given as mean (SD).

### Comparison between microdosimetry and MIRD Dosimetry with ^211^At homogeneously distributed with the thyroid gland

Using the cumulated activity that gives 1 Gy in mean absorbed dose to the thyroid gland based on MIRD formalism in mouse, rat and man, corresponding mean absorbed doses to the follicle cell nucleus based on the microdosimetric calculations were 0.84 Gy (self-contribution of 0.33 Gy (39%) and contribution from the neighbouring follicles of 0.51 Gy), 1.18 Gy (self-contribution of 0.79 Gy (67%) and contribution from the neighbouring follicles of 0.39 Gy), and 0.71 Gy (self-contribution of 0.52 Gy (73%) and contribution from the neighbouring follicles of 0.19 Gy), respectively, for ^211^At homogeneously distributed within the follicle lumens.

## Discussion

When developing a mathematical model of a tissue for dosimetric purposes, it is necessary with simplifications in geometry. For the thyroid, spherical follicles were assumed, although follicles have been described as convex entities of tetrakaidecahedral shape with larger follicles peripherally in the thyroid gland [20]. The present study concerns alpha particles with ranges compared with the species-specific models in the order of the size of one follicle diameter or down to 1/3 of a follicle diameter. For studies on particles with such short ranges (or with very long ranges, when the model does not influence the results at all), we consider spherical models to give realistic results. We also believe that the overall findings and relations between results from different species in this paper are valid and important in translational research.

In the Monte Carlo simulations, only the emitted alpha particles in the ^211^At decay were assumed to deposit energy in the target volume. This assumption was based on the energy released per ^211^At decay for the different radiation types: 6.92 MeV for alpha particles, 0.05 MeV for electrons, and 0.68 MeV for photons [5]. For electrons, the energy released per decay is negligible compared to that of the alpha particles. For photons, the energy released per decay is about 10% of the alpha particles; this fact, combined with the low energy deposition per interaction and long mean free path in tissue, the contribution to the energy deposited in the target volume was neglected. Furthermore, the generation of delta particles and bremsstrahlung from the alpha particle track was not accounted for in the Monte Carlo simulations. The MCNPX code uses the collision stopping power value with energy straggling and multiple scattering algorithms when calculating the energy deposit in the target volume. According to Kellerer and Chmelevsky, the energy deposit for alpha particles in water with kinetic energies less than 10 MeV could accurately be determined without including the delta particles in the calculations for spherical volumes down to a diameter of 1 μm [33]. We, therefore, assumed that neglecting specific calculations of the delta particles would not influence the overall results. Also, the generation of bremsstrahlung could be neglected for heavy charged particles since the generation is inversely proportional to the square of the particle mass [34].

To our knowledge, only one publication has previously dealt with dosimetry for a thyroid model, and that case used the Monte Carlo code CELLDOSE for ^131^I [22]. In that model, a human follicle was represented by a 170-μm diameter sphere consisting of a 150-μm diameter follicle lumen surrounded by a 10-μm layer of thyroid follicle cells. Our human model is similar to that model regarding spherical geometry and the follicle size. However, in the presented study, the influence of size of follicle and cell nucleus was also studied, and specific models for mouse and rat were defined.

When performing calculations for the different species, models were created based on the average thyroid follicle sizes. The mean weight of the thyroid gland for man is approximately 19 g (range 8 to 48 g) [30], for rat approximately 30 mg [31] and approximately 3 mg for mouse [12,17]. There is also a difference in follicle size between the species. In man, the diameter of the follicle varies between 30 and 840 μm [23] with mean values of 160 to 170 μm [21,22]. For rat, the follicle diameter size varies between 20 and 230 μm [19,20] with a mean value of 78 to 88 μm [18-20]. For mouse, only follicular areas have been reported, being 2,000 to 5,000 μm^2^ with a mean value of 2,200 μm^2^ and about 200 follicles per mm^2^[16,17]. This corresponds to a diameter of 53 μm assuming spherical geometry of the follicle. This value might, however, be an underestimation of the true value since all tissue slices were not in the centre of each follicle. The choice of the mean follicle diameters in the mouse, rat and human models was based on these studies. It should be noted the follicle size may change with age [23,35], time of day [19], as well as thyroid disorders [21], and an iodine-rich diet might result in larger follicles [18,20].

The difference in follicle size within the human thyroid results in about 500 times higher 〈z〉 to the follicle cell nucleus in follicles with 30 compared to 500-μm diameter follicle lumen (Figure 3). In man, smaller follicles (diameters < 100 μm) are more common in children (<12 years old) and larger follicles (diameters > 200 μm) more common in adults [23]. For example, 〈z〉 to the follicle cell nucleus is 7 times higher for a 90-μm diameter follicle lumen compared to a 190-μm diameter follicle lumen (both with a 8-μm cell nucleus). This indicates that 〈z〉 could be higher in children. In the mouse model, less than 0.5% of the emitted alpha particles deposit all their kinetic energy within the follicle lumen compared to 7% for the rat model and 45% for the human model. These results indicate that dosimetric parameters cannot readily be translated between animal and human models.

The multiple follicle thyroid model used in this work is a simplification, with a single spherical layer of follicle cells surrounding the central follicle and the neighbouring follicle lumen simulated as a spherical shell around the outer cell layer. The outer follicle lumen had a thickness equal to the diameter of the model used. Inter-follicular tissue was not included in the space between the central follicle and neighbouring follicles and would probably reduce the absorbed dose only to a small extent. In the models created in this work for mouse, rat and human, only one layer of neighbouring follicles will contribute to the energy depositions to the central follicle cell nuclei due to the short range of the alpha particles, justifying the simplification with one surrounding follicle layer. In the model created by Hindie et al., ^131^I in up to 12 layers of follicles contributed to the absorbed dose to the central follicle cells [22]. The self-contribution to the total-absorbed dose from radionuclide in the follicle lumen was 17% for ^131^I [22], compared to 73% for ^211^At as found in the present study. Thus, the single follicle thyroid model, maybe supplied with one surrounding follicle layer, is a simple and realistic model when studying radionuclide emitting particles with ranges up to that of alpha particles, while more follicle layers are needed for particles with longer range.

To our knowledge, no firm data on the biokinetics of ^211^At in the thyroid on the microscopic level exist. We have only found one autoradiographic study demonstrating the distribution of ^211^At within the follicles in rats [10] and a few on the distribution of ^125^I [36,37]. Results from ^125^I in rat show that the concentration of radioiodine early in the uptake phase was higher in the smaller follicles. The distribution within most follicle lumens was homogeneous but heterogeneous in some follicles, usually in the form of a ring peripherally [36,37]. Based on these studies and that ^211^At is supposed not to be bound to thyroglobulin, calculations were done with ^211^At distributed within different source compartments, including heterogeneous distribution within the follicle lumen using concentric spherical surfaces. The results show that 〈z〉 increased with longer surface source radius for the human and mouse models. For the human model, 〈z〉 was zero for short radii of the surface sources due to the range of the alpha particles, while it increased with longer radius. For the rat model, 〈z〉 decreased from a high value at the centre (radius = 0) to a minimum, and then increased up to the maximum radius. For the mouse model, 〈z〉 increased with longer radius. The reason for these differences is the effect of the Bragg peak of the alpha particle, i.e. a higher energy deposition in the end of the track, which should be considered when the follicle lumen radius is similar to the range of the alpha particles.

To our knowledge, microdosimetric determinations have not previously been performed for ^211^At in the thyroid. There are, however, data for ^211^At homogeneously distributed within a cell nucleus calculated by an analytical method [38]. Our results for 〈z〉 and 〈z_1_〉 for follicle cell nucleus diameters of 4, 6 and 8 μm show good agreement (only 2.8% to 3.7% higher values) compared with the published data.

Since iodine is absorbed by the thyroid and has a relatively high atomic number, *Z* = 53, presence of iodine might influence the interaction of the alpha particles within the follicle lumen, leading to a higher energy loss per unit length. In rats, the concentration of stable iodine in the follicle lumen is approximately 0.9% to 2.1% [18]. However, the present results clearly show that the presence of stable iodine in the follicle lumen had little effect on the dosimetric results for mouse and rat models, and the difference in the human model was 6% lower 〈z〉 for an iodine concentration of 2% within the follicle lumen.

The physical properties of the radiohalogens ^211^At, ^131^I and ^125^I vary considerably and result in different mechanisms of action and biological effects on thyroid follicle cells. Alpha particles from ^211^At have a very short range in tissue, resulting in high average LET (approximately 100 keV/μm) and high RBE values. This makes them highly cytotoxic for biological tissue, and cell survival studies have shown that only a few transversals per cell nucleus may inactivate a cell [39,40]. Beta particles from ^131^I main beta spectrum with endpoint energy of 606 keV (89.4% yield) and maximum range of 2.3 mm in tissue [41] result in low average LET (approximately 0.2 keV/μm). It is considered that several thousands of transversals are needed to inactivate a cell [4]. ^125^I emits on average 20 Auger and conversion electrons with ranges from nanometres up to 23 μm and LET values of 4 to 26 keV/μm [42]. When internalised into a cell and especially when incorporated in the DNA, ^125^I can be highly toxic to a cell [43]. We have experimentally compared toxic effects of radionuclides in thyroid follicle cells and shown that the highest effect was obtained for ^211^At, followed by ^123^I (emits Auger and conversion electrons similar to ^125^I), while the least effect was obtained by ^131^I [44].

Usually, MIRD formalism and macrodosimetry have been used to determine mean absorbed dose to the thyroid for ^211^At, assuming homogeneous activity concentration in thyroid tissue [13,14]. To compare if microdosimetry would give other absorbed doses than conventional macrodosimetry, we calculated the absorbed dose to the follicle cell nuclei from the amount of ^211^At homogeneously distributed in the lumens (multiple follicle model) that gives a mean absorbed dose of 1 Gy. Corresponding microscopic data were then 0.84, 1.18 and 0.71 Gy for the mouse, rat and human models, respectively. A similar comparison for ^131^I gave a microdosimetric value of 0.98 Gy to the follicle cells due to the longer ranges of the electrons emitted [22]. Thus, our results show the importance of microdosimetry for non-homogeneous distribution of radionuclides emitting short-ranged particles in a thyroid.

## Conclusions

This study clearly demonstrates differences in energy deposition in the follicle cell nuclei depending on the location of ^211^At in the follicle, and the size of the follicle, the follicle cell and its nucleus, which indicate the importance of species-specific models for dosimetric estimations for ^211^At. The dosimetric parameters for ^211^At in the thyroid cannot readily be translated between species.

## Competing interests

The authors declare that they have no competing interests.

## Authors’ contributions

Both authors designed the study. AJ performed the MC simulations and drafted the manuscript. Both authors contributed to the scientific and intellectual discussion and interpretation of the data and revision of the manuscript. Both authors read and approved the final manuscript.

## Authors’ information

AJ is a PhD student at the Department of Radiation Physics, Sahlgrenska Academy at the University of Gothenburg and has an MSc in medical physics. EFA is a Professor at the Department of Radiation Physics, Sahlgrenska Academy at the University of Gothenburg.
